# The *c4h*, *tat*, *hppr* and *hppd* Genes Prompted Engineering of Rosmarinic Acid Biosynthetic Pathway in *Salvia miltiorrhiza* Hairy Root Cultures

**DOI:** 10.1371/journal.pone.0029713

**Published:** 2011-12-29

**Authors:** Ying Xiao, Lei Zhang, Shouhong Gao, Saengking Saechao, Peng Di, Junfeng Chen, Wansheng Chen

**Affiliations:** 1 Department of Pharmacy, Changzheng Hospital, Second Military Medical University, Shanghai, People's Republic of China; 2 Department of Pharmacognosy, School of Pharmacy, Second Military Medical University, Shanghai, People's Republic of China; 3 Modern Research Center for Traditional Chinese Medicine, Second Military Medical University, Shanghai, People's Republic of China; 4 Department of Plant Sciences, University of California Davis, Davis, California, United States of America; 5 Institute of Chinese Materia Medica, Shanghai University of Traditional Chinese Medicine, Shanghai, People's Republic of China; University of South Florida College of Medicine, United States of America

## Abstract

Rational engineering to produce biologically active plant compounds has been greatly impeded by our poor understanding of the regulatory and metabolic pathways underlying the biosynthesis of these compounds. Here we capitalized on our previously described gene-to-metabolite network in order to engineer rosmarinic acid (RA) biosynthesis pathway for the production of beneficial RA and lithospermic acid B (LAB) in *Salvia miltiorrhiza* hairy root cultures. Results showed their production was greatly elevated by (1) overexpression of single gene, including cinnamic acid 4-hydroxylase (*c4h*), tyrosine aminotransferase (*tat*), and 4-hydroxyphenylpyruvate reductase (*hppr*), (2) overexpression of both *tat* and *hppr*, and (3) suppression of 4-hydroxyphenylpyruvate dioxygenase (*hppd*). Co-expression of *tat*/*hppr* produced the most abundant RA (906 mg/liter) and LAB (992 mg/liter), which were 4.3 and 3.2-fold more than in their wild-type (*wt*) counterparts respectively. And the value of RA concentration was also higher than that reported before, that produced by means of nutrient medium optimization or elicitor treatment. It is the first report of boosting RA and LAB biosynthesis through genetic manipulation, providing an effective approach for their large-scale commercial production by using hairy root culture systems as bioreactors.

## Introduction

**Figure 1:**
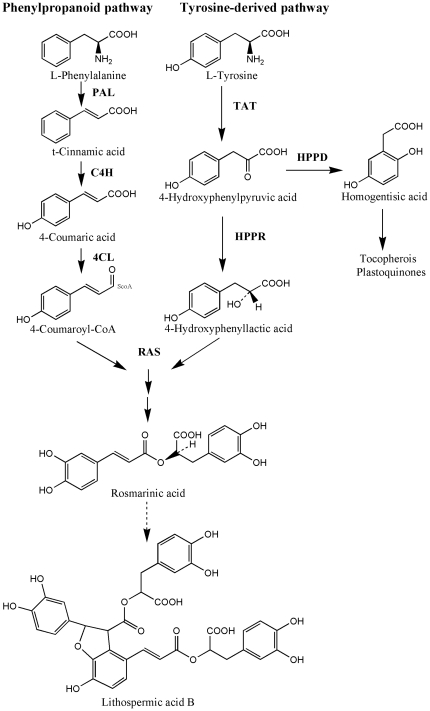
The metabolic pathway leading to RA. PAL; phenylalanine ammonia-lyase, C4H; cinnamic acid 4-hydroxylase, 4CL; hydroxycinnamate coenzyme A ligase, TAT; tyrosine aminotransferase, HPPR; 4-hydroxyphenylpyruvate reductase, RAS; RA synthase, HPPD; 4-hydroxyphenylpyruvate dioxygenase. The dotted line represents a putative biosynthesis process which has not yet been identified.

Rosmarinic acid (alpha-O-caffeoyl-3, 4-dihydroxyphenyllactic acid; RA) is a common hydroxycinnamoyl ester accumulated in the plant species of Boraginaceae and Lamiaceae. In recent years, RA has aroused scientists' interest because it exhibits various biological activities, including antibacterial, anti-oxidative, and antiviral effects [1], as well as has implications toward biofuel feedstock development [2]. These activities make it a potentially useful product for the pharmaceutical, cosmetic and bioenergy industries. The biosynthetic pathway leading to RA has been suggested to involve both the phenylpropanoid pathway and a tyrosine-derived pathway as depicted by Figure 1 [3]. RA formation provides an excellent model to investigate regulatory mechanisms of secondary metabolism because two parallel and presumably concertedly regulated pathways are involved in its biosynthesis [4].

The RA biosynthetic pathway is under strict developmental and environmental control. The enzymology and regulation of RA biosynthesis have been actively investigated by using plant cell cultures. In *Anchusa officinalis* cell suspension cultures, tyrosine aminotransferase (TAT) activity was found positively correlated with the rate of RA biosynthesis during linear growth stage of the culture cycle [5]. In *Lithospermum erythrorhizon* cell suspension cultures, elicitation by methyl jasmonate (MeJA) led to a 10-fold stimulation of RA accumulation, and a strong transient increase in phenylalanine ammonia lyase (PAL) and 4-hydroxyphenylpyruvate reductase (HPPR) activity in the MeJA-treated cells was found to correlate well with the induced RA accumulation [4]. In *Coleus blumei* suspension cultures, a fungal elicitor enhanced RA accumulation ∼3-fold, and the specific activities of PAL and rosmarinic acid synthase (RAS) were coordinately induced [6]. Recently, Song and Wang [7] reported that RNAi-mediated suppression of PAL in *S. miltiorrhiza* caused a marked reduction in RA production. However, the specific role of enzyme(s) involved in activation of the RA pathway remains uncertain.

MeJA, a signal compound, is proposed to play an integral role as a second messenger in the elicitation process leading to the accumulation of secondary metabolites [8], thus it has been commonly used as an elicitor to explore the regulatory mechanisms underlying the biosynthesis of metabolites such as phenolic acid and alkaloids [4], [6], [9]. MeJA-induced plant cell culture provides a responsive model system to profile modulations in gene transcripts and related metabolites accumulations. In our previous study [10], a gene-to-metabolite network was constructed by searching for correlations between the transcripts of RA biosynthetic genes and the accumulations of both RA and its important derivative, lithospermic acid B (LAB) (Figure 1) [11], in MeJA-induced *S. miltiorrhiza* hairy root cultures. This gene-to-metabolite network indicated that *pal*, cinnamic acid 4-hydroxylase (*c4h*), *tat* and *hppr* transcripts were closely correlated with RA and LAB accumulation, implying a role for the corresponding enzymes as metabolic control points for RA and LAB production in *S. miltiorrhiza*. In addition, the successful cloning of *tat*, *hppr*, *c4h* and 4-hydroxyphenylpyruvate dioxygenase gene (*hppd*) from *S. miltiorrhiza* [12]–[14] in our previous study was another great motivation for the exploration of metabolic engineering as an effective approach to appraise the specific role of enzyme(s) in activation of the RA pathway by enhancing rate-limiting steps or by blocking competitive pathways, ultimately leading to an increase in the yield of target compounds (RA and LAB).

Here, we report the introduction of gene constructs containing cDNA clones of respective *tat*, *hppr* and *c4h*, binary *tat* and *hppr*, as well as antisense-*hppd*, driven by the CaMV 35S promoter (*P_35S_*) into *S. miltiorrhiza* hairy roots. The profile of gene transcripts and metabolites accumulations involved in the RA biosynthesis pathway, and phenolic acid production capacities of these engineered hairy root lines were investigated.

## Methods

### Construction of vectors

**Figure 2:**
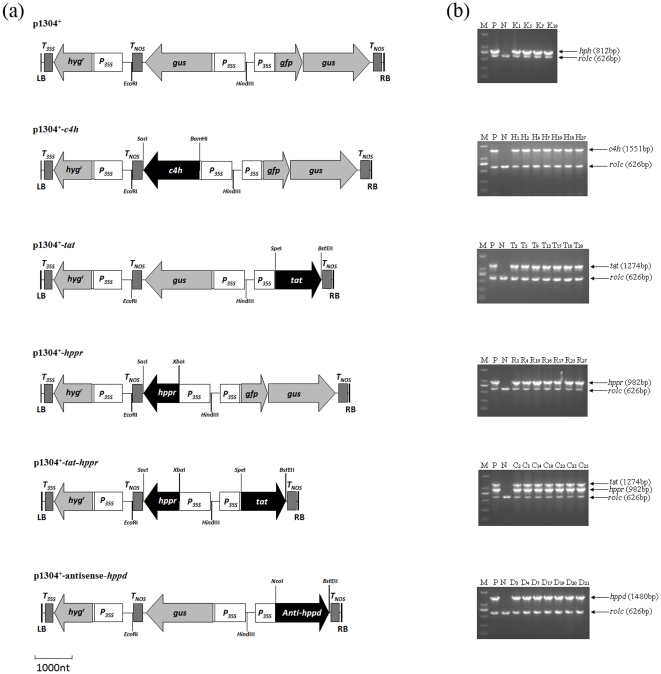
The expression plasmids used in transformation and molecular analyses of transgenic hairy root lines. (a) Schematic representation of transgene expression plasmids. *P_35S_*; CaMV 35S promoter, *hyg^r^*; hygromycin-resistance gene, *gus*; β-glucuronidase coding sequence, *gfp*; green fluorescent protein coding sequence, *T_35S_*; CaMV 35S terminator, *T_NOS_*; nopaline synthase terminator, LB; T-DNA left border, RB; T-DNA right border. Restriction sites and target gene are marked. (b) Representative PCR analyses for the *rolc* gene and the specific genes in transgenic hairy root lines. M; DL-2000 Marker (100–2,000 bp), P; the corresponding engineered bacteria (positive control), N; the wild-type hairy root (negative control), K; vector transformed lines (*ck*), H; single *c4h* transgenic lines, T; single *tat* transgenic lines, R; single *hppr* transgenic lines, D; antisense-*hppd* transgenic lines, C; dual *tat*/*hppr* co-transgenic lines.

The vector pBI121 and pCAMBIA1304 were used to create an intermediate plasmid containing appropriate restriction sites for incorporating not only *c4h*, *tat*, *hppr* and *hppd* gene separately, but also for *tat* and *hppr* together. The pBI121 vector was digested by *Hin*dIII and *Eco*RI, and the 3,033-bp fragment containing the *P_35S_*, β-glucuronidase coding sequence (*gus*) and the nopaline synthase terminator (*T_NOS_*), was recovered. It was then ligated into a *Hin*dIII/*Eco*RI fragment, produced from the double digestion of pCAMBIA1304, to form the recombinant destination vector, designated as p1304^+^. It contained three separate expression cassettes driven by *P_35S_*, and the *hyg^r^* cassette for conferring hygromycin resistance (Figure 2a).

Full length open reading frames for each construct were cloned from cDNA by PCR, according to the published gene sequence information (see GenBank accession numbers in Table S1). Appropriate restriction sites were added to 5′ ends of the primers to amplify each gene coding sequence (see the resulting primer sequences in Table S1). After sequencing confirmation, the obtained DNA sequences of *c4h*, *tat*, *hppr* and *hppd* were digested using their corresponding restriction enzymes (Table S1) and then respectively subcloned into the p1304^+^ vector between *P_35S_* and *T_NOS_*, generating p1304^+^-*c4h*, p1304^+^-*tat*, p1304^+^-*hppr* and p1304^+^-antisense-*hppd* vectors (the bypass gene *hppd* coding sequence was inserted in an inverted orientation to create antisense-*hppd* structure). For *tat*-*hppr* binary expression vector, the *Xba*I/*Sac*I *hppr* gene fragment was subcloned into the p1304^+^-*tat* vector giving plasmid p1304^+^-*tat*-*hppr* (Figure 2a). After PCR and sequencing confirmation, the above five plasmids, together with the construct without the target fragment (i.e. p1304^+^ empty plasmid), were separately introduced into the disarmed *Agrobacterium tumefaciens* C58C1 strain, which carried the pRiA4 of *A. rhizogenes* [15]. After confirmation by PCR, the positive clones were used to transform *S. miltiorrhiza* leaf disc explants.

### Plant transformation and hairy root culture

Transformation of leaf explants from *S. miltiorrhiza* plants was carried out basically following the previously described method [10], with the simultaneous transformation with C58C1 strains as wild-type controls. Hairy roots developed at cut edges 2–3 weeks after co-cultivation were excised and cultured on solid, hormone-free, half-strength B5 medium supplemented with 30 g/l sucrose as the carbon source. All of the culture media contained 100 mg/l hygromycin and 500 mg/l cefotaxime. Root culture clones were maintained at 25°C in the dark and routinely subcultured every 25–30 days. The rapidly growing *hygromycin*-resistant lines with no bacterial contamination were used to establish hairy root lines. About 100 mg of fresh roots (3 cm in length) were inoculated into 150-ml conical flasks containing 40 ml of liquid and half-strength B5 medium, cultured on an orbital shaker (120 rpm) at 25°C in the dark and routinely refreshed the culture medium every 9 days. The fresh weight (fw) of root tissues from flasks of cultures were recorded individually at days 9, 18, 27, 36, and 45 after inoculation. At day 45, the roots were filtered and washed with 10 ml of sterile distilled water and lyophilized, for DNA extraction, RNA extraction and metabolites analysis.

### PCR analysis

Genomic DNA was isolated from hairy root samples using the acetyl trimethyl ammonium bromide (CTAB) method [16]. The DNA was then used in PCR analysis for detecting the presence of the specific genes in transgenic lines. Primer sequences for amplifying these genes (these primers were particularly designed to cover the gene sequence and the vector sequence for detecting exogenous gene transformations) and the expected PCR product size are listed in Table S2. The selectable marker hygromycin resistance gene *hph* (Figure 2a) was used to check the p1304^+^ vector transformants, whereas *Agrobacterium rolc* gene was used to check the transformation of pRiA4 [17]. The PCR reactions were carried out in a total volume of 25 µl comprising 50 ng genomic DNA, 50 mM KCl, 10 mM Tris-HCl (pH 8.3), 1.5 mM MgCl_2_, 200 µM dNTPs, 1.25 units of *Taq* DNA polymerase and 25 pmol of each primer. For PCR analysis, DNA was denatured at 94°C for 3 min followed by 35 cycles of amplification (94°C for 10 s, 58°C for 30 s, 72°C for 1 min) with final extension at 72°C for 5 min.

### GUS assays

The genomic DNA of hairy root samples was used for detecting the presence of the *gus* gene (Figure 2a) by PCR reaction, with primers *gus*-F (5′-GCTGTGCCAGGCAGTTTTAAC-3′) and *gus*-R (5′-ATATCGTCCACCCAGGTGTTC-3′). The amplification condition was the same as those for the specific genes PCR described above. Histochemical localization of GUS activity was performed using 5-bromo-4-chloro-3-indolyl glucuronide (X-Gluc) as the chromogenic substrate. A reaction mixture consisting of 1 mM X-Gluc dissolved in 50 mM sodium phosphate buffer (pH 7.2) was used. Hairy root samples were incubated for 12 h at 37°C and pigments were removed by extraction with 100% ethanol prior to observation [18].

### Real-time quantitative PCR analysis

Total RNAs from hairy root samples were extracted using TRIzol Reagent (GIBCO BRL, Gaithersburg, MD) according to the manufacturer's instruction [19]. The quality and concentration of RNA were examined by ethidium bromide (EB)-stained agarose gel electrophoresis and spectrophotometer analysis. Total RNA was reverse transcribed by using AMV (avian myeloblastosis virus) reserve transcriptase (Takara) to generate cDNA. All of the gene-specific primers were designed according to the conserved region of the corresponding sequences of *S. miltiorrhiza*. Part of the polyubiquitin gene was amplified with primers (5′-ACCCTCACGGGGAAGACCATC- 3′ and 5′-ACCACGGAGACGGAGGACAAG-3′) as a control. Real-time quantitative PCR (RT-qPCR) was performed according to the manufacturer's instructions (Takara) under the following conditions: 1 min pre-denaturation at 95°C, 1 cycle; 10 s denaturation at 95°C, 20 s annealing at a given temperature (set according to the character of the primers used as determined by Primer Premier 5.00 software); and 15 s for collecting fluorescence data at 72°C, 40 cycles. The products of RT-qPCR were subjected to 1.5% agarose-gel electrophoresis and showed an equal-sized band as the predicted product PCR. Quantification of the gene expression was done with the comparative CT method and each data point represents the average of three experiments.

### Analysis of metabolites

Compound extraction and analysis followed the methods described by Yan *et al*. [20] with minor modifications. The dried hairy root sample (50 mg) was ground into powder, comminuted (100 mesh) and extracted twice with 30% ethanol (25 ml) under sonication for 10 min, and then centrifuged at 4500 rpm for 5 min. The supernatant was diluted with distilled water to 50 ml total volume, and the extract solution was then filtered through a 0.2-µm organic membrane before analysis. The concentration of the metabolites was determined by triple-quadrupole mass spectrometer (Agilent 6410, Agilent, Santa Clara, CA) equipped with a pump (Agilent 1200 G1311A, Agilent) and an autosampler (Agilent G1329A, Agilent). Chromatography separation was performed with a Thermo C18 column (150 mm×2.1 mm, i.d., 3.5 µm particle size, Agilent). A mobile phase consisting of acetonitrile: H_2_O (55∶45, v/v) was used, with the flow rate set at 0.3 ml min^−1^ and a 3.5 min run time. Multiple reaction monitoring (MRM) mode was used for the quantification, and the selected transitions of m/z were 359→161 for RA, 717→519 for LAB, 164→147 for L-phenylalanine, 147→102 for t-cinnamic acid, 163→119 for 4-coumaric acid, 180→119 for L-tyrosine, 179→107 for 4-hydroxyphenylpyruvic acid, and 167→123 for homogentisic acid. All standards were purchased from Sigma-Aldrich (St. Louis, MO) [21].

### Statistical analysis

Statistical analysis was performed with SPSS 13.0 software. Analysis of variance (ANOVA) was followed by Tukey's pairwise comparison tests, at a level of *p*<0.05, to determine significant differences between means.

## Results and Discussion

### Gene transformation and confirmation of transgenic lines

Plant expression vectors, including p1304^+^-*c4h*, p1304^+^-*tat*, p1304^+^-*hppr*, p1304^+^-antisense-*hppd* and p1304^+^-*tat*-*hppr*, together with p1304^+^ plasmid as vector control (Figure 2a), were separately introduced into *S. miltiorrhiza* leaf explants by using *A. tumefaciens* C58C1 strain and generated hairy root lines were screened using hygromycin. Some transgenic roots turned brown and aged considerably faster than wild-type root cultures (hairy root lines generated through transformation with blank C58C1 strain, *wt*). These lines were discarded, and the remaining hairy root lines were subcultured for 45 days in hormone-free, half-strength B5 liquid medium, for PCR analysis (Table 1). All of the hairy roots contained the *rolc* gene, which was evidence of transformation by the pRiA4 [17]. To eliminate the interference from *S. miltiorrhiza* endogenous genes, the primers for the integration confirmation of the specific gene(s) into the transformed hairy root genome land on vector sequences (Table S2). PCR analyses confirmed the integration of the exogenous target gene(s) in the tested transgenic lines (Figure 2b). The GUS assays showed that *ck*, *c4h*, *tat*, *hppr* single-gene transformants, as well as antisense-*hppd* lines were positive for the presence of *gus* gene and GUS staining, whereas the *tat*-*hppr* co-transformed lines did not show any detectable PCR product and GUS staining (Figure S1). These results were in accordance with their respective introduced gene constructs (Figure 2a), further demonstrating the success of transgene. These confirmed transgenic lines that also simultaneously showed a good growth capability (Table 1) were selected for biomass growth statistical analysis, as well as further RT-qPCR analysis and metabolites determination.

**Table 1:** Gene constructs and derived root cultures.

	Numbers of established root lines			
T-DNA construct	Total	Antibiotic-resistant	PCR-positive	Established root cultures
p1304^+^-*c4h*	30	18	14	H_1_,H_2_,H_4_,H_7_,H_10_,H_18_,H_27_
p1304^+^-*tat*	25	16	13	T_2_,T_5_,T_9_,T_12_,T_17_,T_18_,T_20_
p1304^+^-*hppr*	27	18	10	R_1_,R_4_,R_10_,R_16_,R_17_,R_25_,R_27_
p1304^+^-antisense-*hppd*	22	10	7	D_3_,D_4_,D_7_,D_17_,D_19_,D_20_,D_21_
p1304^+^-*tat*-*hppr*	27	20	18	C_2_,C_3_,C_14_,C_18_,C_22_,C_23_,C_25_
p1304^+^ vector control (CK)	20	7	4	K_1_,K_5_,K_7_,K_18_

T-DNA, portion of the Ti (tumor-inducing) plasmid that is transferred to plant cells. In addition, three lines transformed with C58C1 were also established, as wild-type control.

### Growth characteristics of transgenic lines

**Figure 3:**
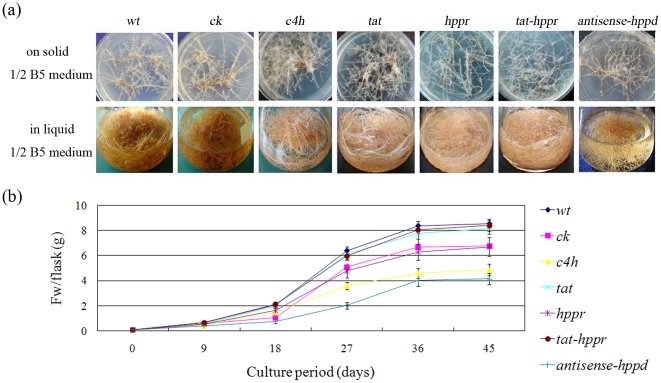
Analyses for the morphology and growth rate in transgenic hairy root lines. (a) Phenotype of developed root lines on solid 1/2 B5 medium for 30 days, and their corresponding root culture in liquid 1/2 B5 medium for 45 days. (b) Time courses of growth of transgenic hairy root lines. Each set of lines corresponds to one transgenic line, and within each set the replicates of independent transgenic line are as depicted by Table 1. Error bars show the standard deviations of independent lines. *wt*; the untransformed hairy root lines (wild-type control), *ck*; empty vector transformed lines (vector control).

The morphology and biomass growth rate of transgenic lines were shown as Figure 3. Significant differences of growth characteristics were detected among independent transformed root lines. All of the *wt*, *tat* and *tat*/*hppr* lines grew fast and vigorously with thick branches whereas other lines (*ck*, *c4h*, *hppr* and antisense-*hppd* lines) grew slowly with slender branches (Figure 3a). Significant differences (*p*<0.05) were found between *wt*, *tat*, *tat*/*hppr* lines and other lines with respect to growth rate, but no significant difference (*p*>0.05) was detected among the tat, *tat*/*hppr* lines and hairy roots generated by blank transformation (*wt*) (Figure 3b). Generally, a fairly high content of secondary metabolites in the tissues is associated with poor growth, and the actual total productivity of secondary metabolites therefore remains low [22]. We found in this study, however, that the growth rate in the high phenolic acid-producing lines was not reduced as compared with those with low phenolic acid production. Line *tat*/*hppr*, for example, which produced the highest level of RA and LAB, grew very rapidly. All types of hairy root lines reached the highest growth rate at day 18 and achieved maximum fw at day 45 after inoculation (Figure 3b).

### Gene expression in transgenic hairy roots

**Figure 4:**
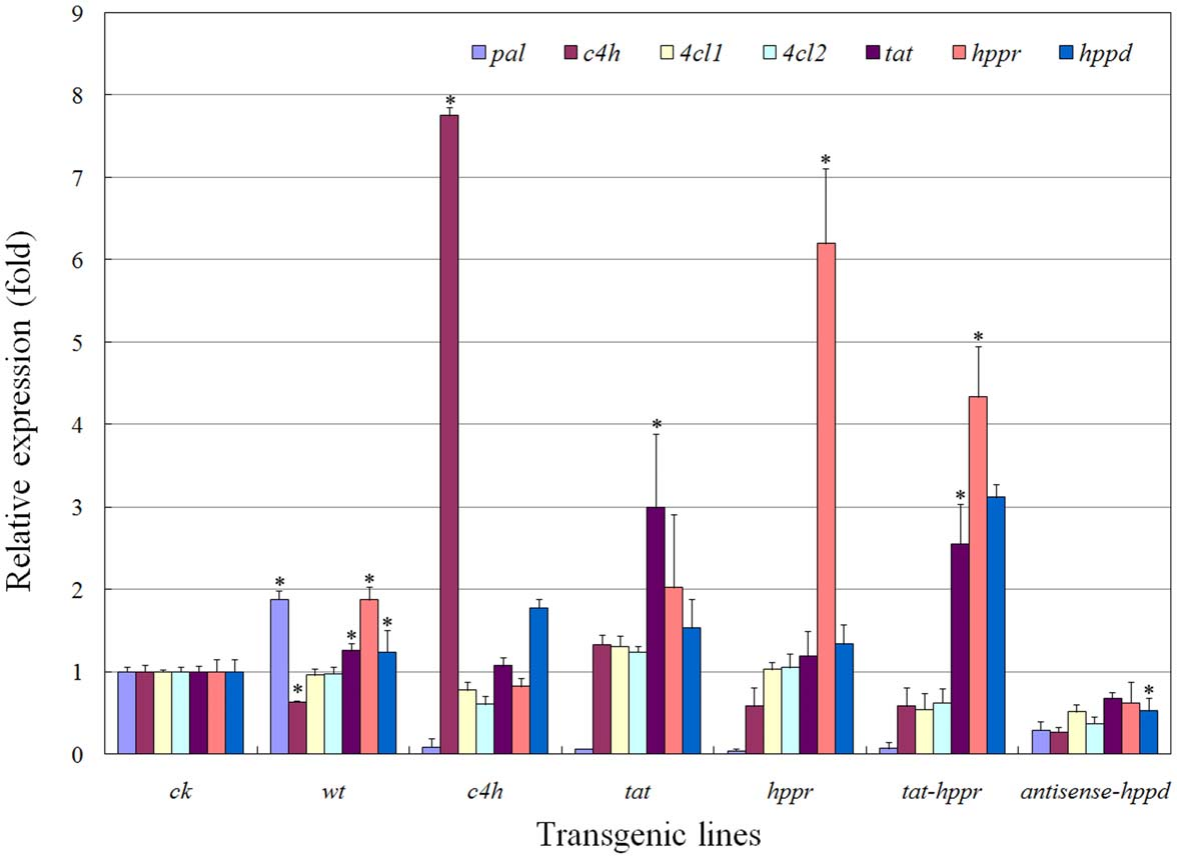
Quantification of the transcripts of RA biosynthetic genes in transgenic hairy root lines. Each set of bars corresponds to one transgenic line, and within each set the different color corresponds to a single gene. The replicates of independent transgenic line are as depicted by Table 1. Error bars show the standard deviations of independent lines. Asterisk indicates that difference is significant at *p*<0.05 compared with vector control. *ck*; empty vector transformed lines (vector control), *wt*; the untransformed hairy root lines (wild-type control).

**Figure 5:**
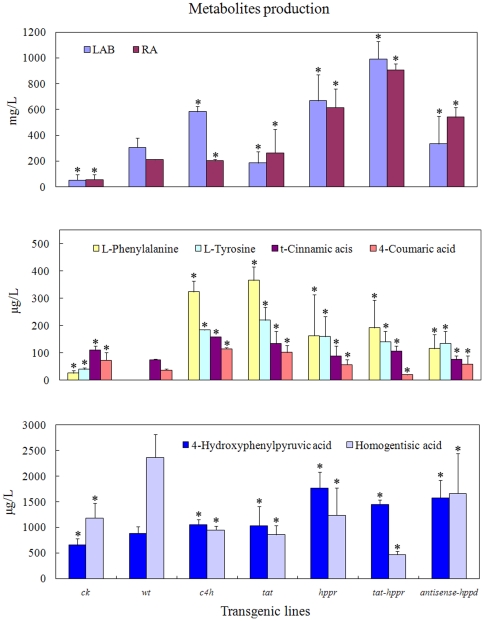
Metabolites production involved in phenolic acids biosynthetic pathway in transgenic hairy root lines. Each set of bars corresponds to one transgenic line, and within each set the replicates of independent transgenic line are as depicted by Table 1. Error bars show the standard deviations of independent lines. Asterisk indicates that difference is significant at *p*<0.05 compared with vector control and untransformed control. *ck*; empty vector transformed lines (vector control), *wt*; the untransformed hairy root lines (wild-type control).

RT-qPCR analysis showed that the expression of the target gene(s) was successfully regulated through genetic manipulation. In comparison with levels of the empty vector transformed control (*ck*), *c4h*, *tat* and *hppr* transcript levels were coordinately enhanced (approx. 7.7, 3.0 and 6.2-fold, *p*<0.05) in their respective overexpression lines. The transcript levels of *tat* and *hppr* were up-regulated ∼2.5 and ∼4.3-fold (*p*<0.05) by simultaneous introduction, whereas *hppd* transcript was reduced ∼50% (*p*<0.05) by antisense approach (Figure 4). In addition, gene transcript fluctuations between *ck* and *wt* lines were generally observed, indicating the introduction of vector p1304^+^ made modulations in gene transcript profiles of *S. miltiorrhiza* hairy roots, a similar phenomenon was found in vector pKANNIBAL transformation of *S. miltiorrhiza* plantlets [7]. Meanwhile, the transcript profile analysis showed these specific transgenic manipulations also variably affected the transcript level of several other RA biosynthetic genes, implying the occurrence of regulatory crosstalk. For example, *hppd* suppression simultaneously down-regulated the overall gene transcripts when compared with *ck*, and *pal* transcript level was generally decreased by these bioengineering manipulations (Figure 4), which subsequently resulted in the high L-phenylalanine (the precursor of PAL) accumulation in the corresponding engineered lines (Figure 5). The transcript crosstalk phenomenon among RA synthesis-related genes has been widely observed in previous studies [7], [23]–[25], possibly occurring via a feedback loop involving coordinated regulation of a series of pathway enzymes [23].

### Metabolites accumulation in transgenic hairy roots

The profiles of metabolites involved in the RA biosynthetic pathway in transgenic hairy root lines were determined by triple-quadrupole mass spectrometer. Results revealed a broad and dynamic reconfiguration of metabolic flux in engineered hairy root cultures that was similar to modulations in transcript profiles (Figure 5). All metabolic pathway strategies significantly enhanced target phenolic acids (RA and LAB) accumulations compared with *ck* (*p*<0.05), despite the lower transcript level of *pal* that was generally found in these engineered root lines (Figure 4). Previously *pal* was suggested to possess great importance to phenolic acids synthesis in *S. miltiorrhiza* [7], [10], [26], our result presented here implies that the *pal* gene plays an important role but not as a bottleneck in the RA biosynthesis pathway. The activation in the sequential biosynthetic steps could effectively compensate for the reduced flux resulted from the disruption of *pal*.

Interestingly the introduction of empty vector p1304^+^ into *S. miltiorrhiza* hairy roots greatly inhibited RA and LAB biosynthesis in comparison with *wt*. However, their accumulations increased following introduction of the target genes. Comparison of metabolites profile among these plant tissues demonstrated a strong correlation between 4-hydroxyphenylpyruvic acid accumulation and their capacity to produce phenolic acids. Elevated 4-hydroxyphenylpyruvic acid accumulation was widely detected in the highly productive transgene lines (compared with *wt*, *p*<0.05), while the lower ability of *ck* lines to accumulate phenolic acids may result from the lower 4-hydroxyphenylpyruvic acid accumulation (compared with *wt*, *p*<0.05) (Figure 5). For the unique role of 4-hydroxyphenylpyruvic acid not only as a product of TAT, but also as a co-substrate of HPPR and HPPD, this finding suggests that 4-hydroxyphenylpyruvic acid may represent regulatory bottlenecks in phenolic acid biosynthesis, or perhaps points of contact between separate metabolic channels, and that its accumulation probably has a positive influence on the flow of metabolites through the pathway. However, this remains to be investigated by further studies for stably producing and more efficient transformants.

C4H catalyses the second reaction of the phenylpropanoid pathway and has been one of the most studied cytochrome P450 enzymes in plants, exhibiting a key role in the synthesis of the phenylpropanoid polymer lignin [23], [27]–[29]. However, its effects on phenolic acids synthesis have not been as extensively studied. Here we observed overexpression of *c4h* activated two parallel pathways of RA biosynthesis, resulted in increased t-cinnamic acid and 4-coumaric acid accumulation in the phenylpropanoid pathway, increased L-tyrosine and 4-hydroxyphenylpyruvic acid in the tyrosine-derived pathway, and lastly the large production of end-product phenolic acids. The content of LAB in *c4h* transgenic lines was enhanced to 584 mg/liter, which was 11.1-fold of that in *ck* (52.7 mg/liter), and 1.9-fold of *wt* (307 mg/liter). Additionally, *c4h* transformation produced comparable RA concentration (201 mg/liter) with that in *wt* (211 mg/liter), which was ∼3.6-fold more than *ck* (56.1 mg/liter).

Overexpression of the tyrosine-derived pathway gene *tat* and *hppr*, either separately or simultaneously, activated the tyrosine-derived pathway, stimulating L-tyrosine and 4-hydroxyphenylpyruvic acid accumulation, and led to enhanced phenolic acids production compared with *ck*. However, the phenolic acids' production levels were highly variable. For example, the *tat* single-gene transgenic hairy root lines accumulated phenolic acids only at similar levels to those of *wt*, likely due to its relatively lower expression level (only 3 times of *ck*) (Figure 4), which was not sufficient to ensure maximal activity for promoting phenolic acids biosynthesis. In contrast, single *hppr* transgenic lines presented much higher RA (616 mg/liter) and LAB (669 mg/liter) production. As expected, the greatest abundance of target compounds was found in *tat*-*hppr* double gene-transformation lines, which produced RA and LAB with the content of 906 and 992 mg/liter, values that are more than 4.3 and 3.2 times that in *wt* lines, and over 16.1 and 18.8 times that in *ck* lines, respectively. Notably, although possessing a relatively low *tat* and *hppr* transcript level compared with the single gene transformed lines, the *tat*-*hppr* co-transformed lines produced the highest level of desirable metabolites. This result implies that the high phenolic acids level was associated with a coordinative effect of both *tat* and *hppr* transgenes. Meanwhile, *tat*-*hppr* transgenic lines was fascinatingly observed to possess the lowest accumulation level of homogentisic acid, a compound involved in the competitive branch of RA biosynthesis and produced by HPPD from 4-hydroxyphenylpyruvic acid precursor. This most likely suggests a metabolic flux shift from the homogentisic acid-branch towards the RA pathway. The strong pulling force from TAT and HPPR greatly boosted the overall RA pathway, and subsequently directed the flux from the homogentisic acid pool to restore the metabolic balance, leading to decreased homogentisic acid levels and increased phenolic acid accumulations. It was concluded that transgenic hairy roots harboring both *tat* and *hppr* possessed an increased flux in the phenolic acids biosynthetic pathway that enhanced RA and LAB yield, which was more efficient than that harboring only one of the two genes. A similar “push-pull” strategy has been reported in several previous studies, with a significant increase in provitamin A, limonene and artemisinin yields in plants such as *Oryza sativa, Lavandula latifolia* and *Artemisia annua,* respectively [30]–[32], as well as enhancement of scopolamine, tropane alkaloid and terpenoid indole alkaloid accumulation in hairy roots of *Hyoscyamus niger*, *Anisodus acutangulus* and *Catharanthus roseus* [33]–[35]. Currently, an increasing number of researchers are embracing this strategy as an approach to generate plants with more ambitious phenotypes instead of introducing single genes [36].

HPPD transforms 4-hydroxyphenylpyruvate to homogentisate, competing for the same substrate as HPPR. Homogentisate is then transformed to tocopherols, commonly known as vitamin E [37]. Because of the high nutrition value of vitamin E, previous investigation in HPPD usually focused on its contribution to the production of vitamin E [38]–[40]. Here, we firstly examined the role of HPPD as a side-branch enzyme of the RA biosynthesis pathway for phenolic acids accumulations. Suppression of *hppd* directly increased accumulation of its substrate (4-hydroxyphenylpyruvic acid), in comparison with *ck*, indicating this genetic manipulation successfully inhibited the substrate competition come from the bypass way. However, homogentisic acid (the catalysate of HPPD) was also enhanced by *hppd* suppression. This could be associated with the widespread suppression effect of antisense-*hppd* on the biosynthetic genes, such as shown in Figure 4. It may be envisaged that the lower gene expression-causing flux limitation upon the downstream of homogentisic acid pathway would make more homogentisic acid accumulation. The *hppd*-suppressed hairy roots produced higher levels of RA (542 mg/liter) and LAB (334 mg/liter) than both the *wt* and *ck* lines (*p*<0.05), which was in accordance with our anticipation that HPPD activity was of great importance to the synthesis of phenolic acids in *S. miltiorrhiza* [10]. However, the *hppd* engineered roots accumulated more RA than LAB, which contrasts with the native distribution of phenolic acids in *S. miltiorrhiza*, where the content of LAB was found to be much higher than that of RA [41]. This probably resulted from the directional engineering effect upon the specific RA pathway rather than LAB, demonstrating the power of genetic engineering to reprogram phenols metabolism and distribution in plant culture.

Above all we conclude as follows: (1) By using genetic transformation, the present study demonstrates previous gene-to-metabolite network described role of those enzymes as critical metabolic control points for the production of target phenolic acids in *S. miltiorrhiza* hairy root cultures. Furthermore, phenolic acid production capacities of these engineered root lines paralleled their corresponding gene-to-metabolite correlation coefficients drawn by previous study. For instance, the correlation coefficient corresponding to *hppr* was much more than *tat*. That is, *hppr* transgene lines exhibited higher level of phenolic acids than their *tat* counterparts. (2) Compared with those MeJA-treated lines (494 mg/liter RA and 1585 mg/liter LAB production) [10], *hppr* single-gene, *tat*-*hppr* double-gene transformants, as well as antisense-*hppd* lines reported here were all observed to accumulate more RA but less LAB. On the one hand, these results fully display the power of genetic engineering for direct regulation of the specific RA pathway versus elicitor induction. However, on the other hand the results also prompt us to speculate combining transgenic technology with elicitor treatments as another feasible strategy to further improve phenolic acids yield in *S. miltiorrhiza* hairy roots in the near future [42], [43]. (3) It appears that the pulling force from the phenylpropanoid pathway involved genes, such as *c4h*, play a more important role in stimulating LAB accumulation, whereas genes involved in the tyrosine-derived pathway, e.g. *tat, hppr, hppd*, make a greater contribution to RA biosynthesis regulation. At the extreme, *hppd* engineered hairy roots contained much more RA than LAB, which completely contrasted with their natural distribution in *S. miltiorrhiza*. (4) Gene activation of the RA pathway was found to not only boost RA biosynthesis, but also promote accumulation of its derivative LAB. This result further strengthens our previously reported hypothesis that RA is a precursor leading to LAB synthesis [11].

With our ever increasing knowledge of complex plant metabolic networks involved in the production of biologically active plant compounds [44], a deeper understanding of the regulatory system governing secondary metabolism attracts particular interest and could eventually allow for the possibility of successful metabolic engineering of target compound biosynthesis [9]. In the current study, by using attractive targets enabled by previously described gene-to-metabolite network, we successfully engineered the RA biosynthesis pathway for the production of beneficial RA and its derivative LAB in *S. miltiorrhiza* hairy root cultures, shedding light on how to effectively increase the end products of secondary metabolic pathways by more accurate and appropriate genetic engineering strategies. Moreover, it is the first report of boosting the RA biosynthesis pathway through genetic manipulation. Co-expression of *tat*/*hppr* dramatically activates the RA pathway, produces high concentration of RA (906 mg/liter), a value more than that produced by means of nutrient medium optimization or elicitor treatment reported previously, in plant cell cultures such as cell suspensions of *L. erythrorhizon* [4], *C. blumei* [6], *S. miltiorrhiza* [26], and *Lavandula vera MM* [45], [46], as well as hairy roots of *S. miltiorrhiza* [10], [11], [20], *Hyssopus officinalis* [47], *Salvia officinalis* [48], and *Coleus forskohlii* [49]. The current study provides an effective approach for commercially large-scale production of RA by using the *S. miltiorrhiza* hairy root systems as bioreactors.

## Supporting Information

Table S1 PCR primers of the coding sequences of *c4h*, *tat*, *hppr* and *hppd* genes.
(DOC)

Table S2 PCR primers used for detecting the specific genes in transgenic lines.
(DOC)

Figure S1 **The transgenic hairy roots were verified by GUS assays.** (a) PCR analysis of hairy root DNA using the primers to amplify a 425-bp fragment of the *gus* gene. M; DL-2000 Marker (100–2,000 bp), P; the pCAMBIA1304 plasmid (positive control), N; the wild-type hairy root (negative control). (b) Corresponding GUS histochemical staining of transgenic hairy roots.
(TIF)
