# Supplementary material for: The c4h, tat, hppr and hppd Genes Prompted Engineering of Rosmarinic Acid Biosynthetic Pathway in Salvia miltiorrhiza Hairy Root Cultures
Source: PLoS One. 2011 Dec 29;6(12):e29713. doi: 10.1371/journal.pone.0029713 (PMC3248448; doi:10.1371/journal.pone.0029713)
Supplement: Table S1 — PCR primers of the coding sequences of c4h, tat, hppr and hppd genes. (DOC) [file pone.0029713.s001.doc]

**Table S1. PCR primers of the coding sequences of *c4h*, *tat*, *hppr* and *hppd*** genes

| Gene/  GenBank accession numbers | Primer sequences (5’→ 3’) |
| --- | --- |
| *c4h*  Q43240 | *Bam*HI  Upstream: AAGGATCCATGGATCTCCTCCTCCTCGAG |
|  | *Sac*I  Downstream: GCGAGCTCTCAAAATGATCTCGGCTTCAA |
| *tat*  DQ334606 | *Spe*I  Upstream: GGACTAGTATGGAGTTGCAGAATCCAGCG |
|  | *Bst*EII  Downstream: AAGGTNACCTTAGTAGGAGTGCCGTTCACA |
| *hppr*  DQ099741 | *Xba*I  Upstream: GGTCTAGAATGGAGGCGATCGGTGTTCTG |
|  | *Sac*I  Downstream: TTGAGCTCTCAAACCACAGGTGTTAACAG |
| *hppd*  EF157837 | *Bst*EII  Upstream: ATGGTNACCATGACAAGTATACATCTCTCT |
|  | *Nco*I  Downstream: TTCCATGGTCACGTCGCTGCTGCTCTCGT |
