# Supplementary material for: The c4h, tat, hppr and hppd Genes Prompted Engineering of Rosmarinic Acid Biosynthetic Pathway in Salvia miltiorrhiza Hairy Root Cultures
Source: PLoS One. 2011 Dec 29;6(12):e29713. doi: 10.1371/journal.pone.0029713 (PMC3248448; doi:10.1371/journal.pone.0029713)
Supplement: Table S2 — PCR primers used for detecting the specific genes in transgenic lines. (DOC) [file pone.0029713.s002.doc]

**Table S2**. PCR primers used for detecting the specific genes in transgenic lines

| Specific gene | Primer sequences (5’→ 3’) | PCR product size (bp) |
| --- | --- | --- |
| *c4h* | *Bam*HI  F*c4h*: GGACTCTAGAGGATCCATGGATCTCCTCC | 1551 |
|  | *Sac*I  R*c4h*: GATCGGGGAAATTCGAGCTCTCAAAATGA |  |
| *tat* | *Spe*I  F*tat*: GATCGGGGAAATTCACTAGTATGGAGTTG | 1274 |
|  | *Bst*EII  R*tat*: AAATTCGAGCTGGTNACCTTAGTAGGAGTG |  |
| *hppr* | *Xba*I  F*hppr*: AGAACACGGGGGACTCTAGAATGGAGGCG | 982 |
|  | *Sac*I  R*hppr*: GATCGGGGAAATTCGAGCTCTCAAACCAC |  |
| *hppd* | *Bst*EII  F*hppd*: AAATTCGAGCTGGTNACCATGACAAGTATA | 1480 |
|  | *Nco*I  R*hppd*: GGACTCTTGACCATGGTCACGTCGCTGCT |  |
| *hph* | F*hph*: cgatttgtgtacgcccgacagtc | 812 |
|  | R*hph*: CGATGTAGGAGGGCGTGGATATG |  |
| *rolc* | F*rolc*: CTCCTGACATCAAACTCGTC | 626 |
|  | R*rolc*: TGCTTCGAGTTATGGGTACA |  |
