# Supplementary figures and images for: The c4h, tat, hppr and hppd Genes Prompted Engineering of Rosmarinic Acid Biosynthetic Pathway in Salvia miltiorrhiza Hairy Root Cultures
Source: PLoS One. 2011 Dec 29;6(12):e29713. doi: 10.1371/journal.pone.0029713 (PMC3248448; doi:10.1371/journal.pone.0029713)

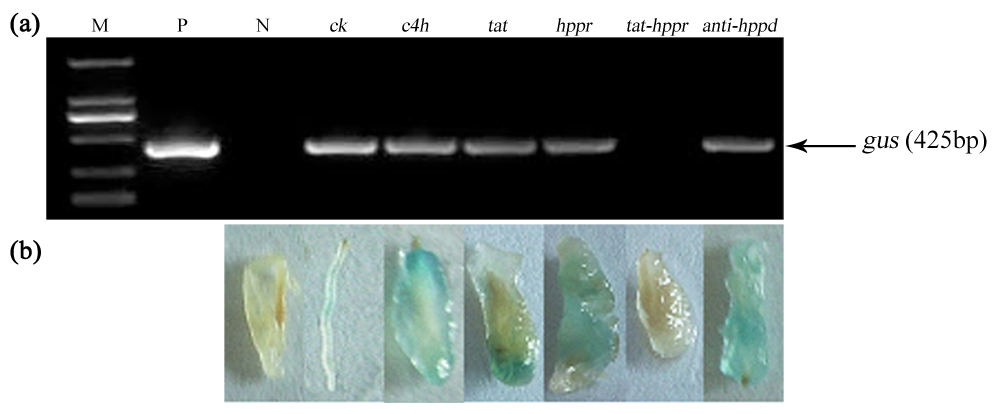

Supplement: Figure S1 — The transgenic hairy roots were verified by GUS assays. (a) PCR analysis of hairy root DNA using the primers to amplify a 425-bp fragment of the gus gene. M; DL-2000 Marker (100–2,000 bp), P; the pCAMBIA1304 plasmid (positive control), N; the wild-type hairy root (negative control). (b) Corresponding GUS histochemical staining of transgenic hairy roots. (TIF) [file pone.0029713.s003.tif]
